# The prevalence of psychiatric disorders in patients with Multiple Sclerosis in Saudi Arabia: A cross-sectional multi-centered study

**DOI:** 10.1192/j.eurpsy.2023.1837

**Published:** 2023-07-19

**Authors:** A. Alserihi, A. Alswat, B. Altirkistani, O. Baeshen, E. Alrushid, J. Alkhudair, O. Wadaan, A. Aldbas, A. Alsaleh, Y. Al Malik, A. Abulaban, S. Makkawi

**Affiliations:** 1 College of Medicine, king Saud bin Abdulaziz University for Health Sciences; 2 College of Medicine, king Saud bin Abdulaziz University for Health Sciences, Jeddah, Saudi Arabia, Jeddah; 3 College of Medicine, king Saud bin Abdulaziz University for Health Sciences; 4 Department of Medicine, King Abdulaziz Medical City, National Guard Health Affairs, Riyadh; 5 Department of Medicine, King Abdulaziz Medical City, National Guard Health Affairs, Jeddah, Saudi Arabia

## Abstract

**Introduction:**

Multiple sclerosis is considered one of the leading causes of neurological non-traumatic disability among young people. Given the chronic progressive nature of the disease, psychiatric disorders are more prevalent among those patients as reported in the literature; however, the data in Saudi Arabia is lacking.

**Objectives:**

The study aimed to estimate the prevalence of psychiatric disorders among MS patient in Saudi Arabia.

**Methods:**

This was a cross-sectional multi-centered study, including patients with multiple sclerosis. Participants were interviewed and asked to fill a validated survey that consisted of demographics, Patient Health Questionnaire-9 (PHQ-9), and Generalized Anxiety Disorder-7 (GAD-7) questionnaire. Descriptive statistics were performed, and the analysis were made using Chi-square, Fisher’s exact, and ANOVA tests as appropriate.

**Results:**

A total of 192 participants were included in the study. Based on a cutoff score of > 10 in the GAD-7 and PHQ-9 scales, the prevalence of anxiety was 26.1% (n-50), with majority of the participants having minimal anxiety (40%); meanwhile, the prevalence of depression was 42.7% (n=82), and most of them had mild depression (30%). [table 1, image 1 and 2] Females participants significantly scored higher in GAD-7 and PHQ-9 compared to males (p-value= 0.0376 and 0.1134, respectively). [table 2 and 3] In addition, no significant association was detected between functional disability (EDSS score) and the prevalence of anxiety and depression.

**table 1:** 

**Items**	
**GAD 7 score, Mean (SD)**	6.5 (±4.9)
**GAD 7, N (%)**	
Fit for Anxiety (>10) *	50 (26%)
Not Fit for Anxiety (<10) *	142 (74%)
**PHQ 9 score, Mean (SD)**	8.8 (±6.1)
**PHQ 9, N (%)**	
Fit for Depression (>10) *	82 (42.7%)
Not Fit for Depression (<10) *	110 (57.3%)

**table 2:** 

	**Fit for Anxiety (n=50)**	**Not Fit for Anxiety (n=142)**	**P Value**
**Gender, N (%)**			
- **Male**	10 (20%)	51 (35.9%)	0.0376
- **Female**	40 (80%)	91 (64.1%)

**table 3:** 

**N(%)**	**Fit for Depression (n=82)**	**Not Fit for Depression (n=110)**	**P Value**
**Gender, N (%)**			
- **Male**	21 (25.6%)	40 (36.4%)	0.1134
- **Female**	61 (74.4%)	70 (63.6%)

**Image:**

**Figure:**
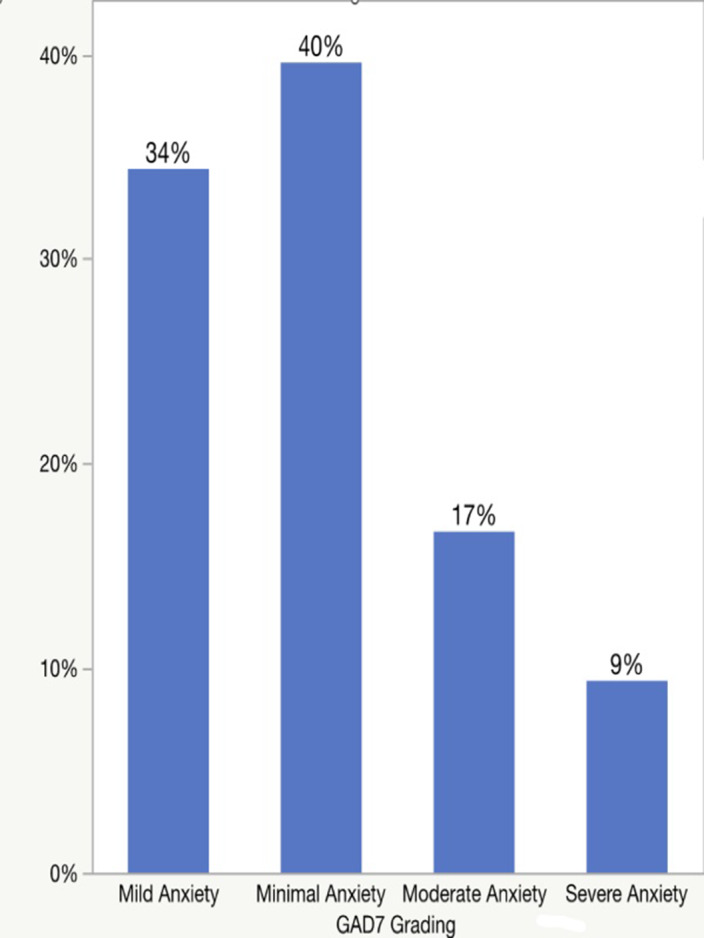

**Image 2:**

**Figure:**
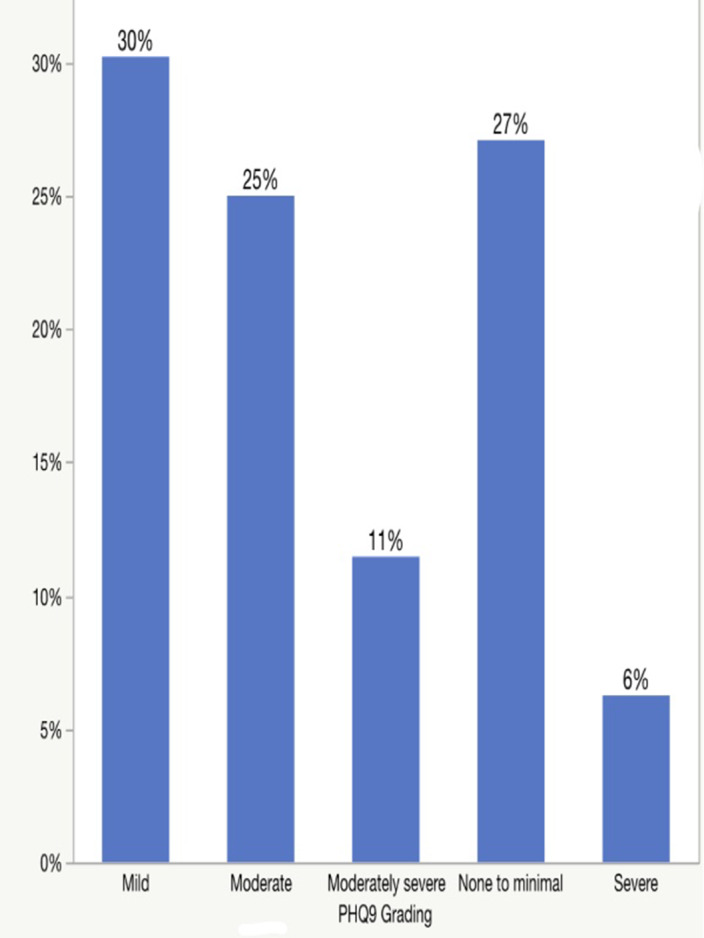

**Conclusions:**

This study reported high level of anxiety and depression among MS patients, with females being more affected. Since these co-morbid disorders could affect the disease course negatively, screening is of paramount significance.

**Disclosure of Interest:**

None Declared

